# Effects of Freeze–Thaw Event on Microbial Community Dynamics During Red Clover Ensiling

**DOI:** 10.3389/fmicb.2019.01559

**Published:** 2019-07-09

**Authors:** Zhihao Dong, Junfeng Li, Lei Chen, Siran Wang, Tao Shao

**Affiliations:** Institute of Ensiling and Processing of Grass, College of Agro-grassland Science, Nanjing Agricultural University, Nanjing, China

**Keywords:** freeze–thaw event, red clover, microbial community, fermentation quality, next-generation sequencing

## Abstract

Freezing damages in forages represents a major economic loss to agriculture. This study was conducted to investigate the effects of freeze–thaw (FT) event on microbial community dynamics of red clover silage. Results showed that the FT-treated material displayed higher proportions of *Weissella* and aerobic bacteria, while lower *Pantoea* and *Enterobacter* compared with the control material. The FT event promoted the development of *Lactobacillus* in silage microflora, inducing more intense lactic fermentation after an initial short lag. The aerobic bacteria were suppressed immediately after the onset of ensiling. Microbiomes of the two silages tended to be almost similar after 2 days of ensiling. However, a small number of aerobic bacteria tended to revitalize in the FT silage with prolonged ensiling time, indicated by apparent abundances of *Acinetobacter* and *Pseudomonas* at the end of ensiling. The results obtained here suggest that the FT event could promote the development of *Lactobacillus* during ensiling and the control of aerobe revitalization need to be concerned with silages made from the freeze-damaged forages.

## Introduction

In boreal and temperate regions, forage crops are frequently exposed to freezing temperature during autumn, spring, and mild winter. This leads to reduction in field productivity and, particularly, problem in forage utilization due to altered physical properties after thawing (Koponen et al., 2006). Direct ensiling could be an advisable choice as it avoids the waste resulting from the aerobic spoilage in field. Ensiling is a conservation method based on spontaneous lactic acid fermentation under anaerobic condition. It could be expected that the freeze–thaw (FT) stress would cause physical damages to plant, promote nutrient release from plant cell (Phalakornkule et al., 2017) and consequently benefit the development of lactic acid bacteria (LAB) during ensiling (Charmley et al., 1997). However, on the other hand, the damaged forages may also serve as an open culture medium allowing vigorous growth of various bacteria. Although it is known that a great majority of these bacteria are aerobes and will be suppressed after anaerobiosis is achieved during ensiling, some undesirable facultative aerobes, such as enterobacteria, might remain active during ensiling (McDonald et al., 1991). This brings the uncertainties of silage fermentation quality made from the FT-damaged forages. Evaluation of potential of the FT-damaged forages in silage making would be important as to supporting the current shifts toward sustainable and low-cost agricultural systems.

Red clover (*Trifolium pratense* L.) is a leading legume forage widely cultivated in boreal and temperate regions. Compared to alfalfa (*Medicago sativa* L.), red clover is characterized by rapid spring establishment and superior performance on acid and wet soils (Bertrand et al., 2016). However, as a promising forage crop, characterization of microbial communities in red clover silage is far behind from those in other forage species, such as alfalfa (Zheng et al., 2017; Yang et al., 2019) and whole crop corn (Keshri et al., 2018; Ni et al., 2018). Understanding of the microbial communities involved in the ensiling process would provide insight into approaches to improve the conservation of red clover.

Many techniques have been developed to describe microbial communities in silage. These include culture-based techniques (Ercolini, 2004), real-time PCR (Stevenson et al., 2006), and denaturing gradient gel electrophoresis (Ni et al., 2017a). In general, none of these techniques are powerful enough to identify species present in low abundance, some of which may nevertheless be critical for optimal fermentation (Gharechahi et al., 2017). The current next-generation sequencing (NGS) technique allows a much greater level of resolution than has been available in the past and therefore widely used in various micro-ecological environments (Liu et al., 2019). The objective of this study was to characterize the effects of freeze–thaw (FT) event on microbial community dynamics and fermentation quality of red clover silage with the application of NGS high-throughput sequencing.

## Materials and Methods

### Silage Preparation and Experiment Design

Red clover was cultivated in experimental field of Nanjing Agricultural University, Nanjing, Jiangsu, China (N31°14″, E118°22″), and harvested on November 12, 2017. The growth stage was at late bud to early bloom. The stubble was about 10 cm above ground level. After harvest, red clover was immediately transferred to laboratory and chopped to a theoretical length of 1∼2 cm with a forage chopper. The chopped red clover was equally divided into two groups after thoroughly mixing. The first group was untreated control. The second group was frozen in a commercial refrigerator (−20°C) for 2 h and then thawed for 1 h at room temperature (10∼16°C). This FT process was repeated three times. After that, these forages (approximately 500 g for each bag) were picked into thick polyethylene bags (200 μm thick), vacuumed, and stored in a light-blocking box at room temperature. CK is abbreviation of untreated control silage and FT is FT treated silage. These silos were opened after 1, 2, 4, 8, and 30 days of ensiling, respectively. There were three replicates for each treatment per day. After opening, fermentation quality, microbial population, and microbial community were determined.

### Chemical Analyses

Approximately 35 g silage sample was extracted in 70 ml of deionized water at 4°C for 24 h to obtain the extract. The pH of the extract was measured with an electrode pH meter (HANNA pH 211, Hanna Instruments, Italy). The extract was centrifuged for 10 min at 10,000 × *g*, and the supernatant was reserved for organic acid (including lactic, acetic, propionic, butyric acid, and ethanol) analysis. The quantification of organic acid was conducted using an Agilent 1260 HPLC system equipped with a refractive index detector (Carbomix H-NP5^®^ column, 2.5 mM H_2_SO_4_, 0.5 mL/min). Nitrogen compounds including free amino acid (FAA-N), ammonia (NH_3_-N) and non-protein nitrogen (NPN) were measured in the extracts using the method presented by Li et al. (2018) and the results were all expressed as g/kg of total nitrogen (TN).

The plant tissue damage degree (TTD) was measured in the ensilage materials according to the method of Lee et al. (2009). Ensilage material and silage samples were freeze-dried for 48 h to determine dry matter (DM) content. TN was determined with the method of Association of Official Analytical Chemists [AOAC] (1990). The water-soluble carbohydrates (WSC) were quantified as the method of Dong et al. (2017).

### Microbial Analysis by Culture-Based Method

Ten grams of sample was blended with 90 mL of sterilized water and serially diluted from 10^–1^ to 10^–9^ in sterilized water. The LAB was counted on de Man, Rogosa and Sharpe agar medium, incubated in an anaerobic incubator at 37°C for 3 days. Yeasts were enumerated on potato dextrose agar after aerobic incubation at 28°C for 2 days. The yeasts were distinguished from moulds and other bacteria by colony appearance and cell morphology. The aerobic bacteria were estimated by using nutrient agar after aerobic incubation at 30°C for 24 h.

### Microbial Diversity Analysis

#### Microbial DNA Isolation

For the molecular analysis of the microbial communities, genomic DNA was extracted through the following steps: 10 g of silage sample was mixed with 90 mL of sterile 0.85% NaCl solution, and treated with a table concentrator at 120 r/m for 2 h. After filtering with carbasus, and the liquor was centrifuged at 10,000 r/m for 10 min at 4°C. The supernatant was discarded, and the deposit was suspended in 1 mL of sterile 0.85% NaCl solution. The liquor was centrifuged at 12,000 r/m for 10 min at 4°C, and the supernatant was discarded. The pellet was used for DNA extraction. Total DNA were extracted with TIANamp Bacteria DNA isolation kit. The agarose gel electrophoresis and Nano Drop 2000 118 UV-vis spectrophotometer (Thermo Fisher Scientific, Wilmington, United States) were used to check the quality of DNA after extraction.

#### PCR Amplification

Approximately 10 ng of DNA isolated from each sample was used for amplification. The primers were 338F (5′-ACTCCTACGGGAGGCAGCAG-3′) and 806R (5′-GGACTACHVGGGTWTCTAAT- 3′) targeting the V3-V4 regions of 16S rRNA genes.

The PCR reaction system consisted of 4 μL of 5 × FastPfu Buffer, 2 μL of 2.5 mmol/L dNTPs, 0.8 μL of each primer (5 μM), 0.4 μL of FastPfu Polymerase, and 10 ng of template DNA. These reactions were performed by thermocycler PCR system (GeneAmp 9700, ABI, United States) under the following condition: a prior denaturation at 95°C for 30 min, followed by 27 cycles of denaturation 30s at 95°C, annealing at 55°C for 30 s, elongation at 75°C for 45 s, and a final extension at 72°C for 10 min. The PCR products were extracted by 2% agarose gel and purified using the AxyPrep DNA Gel Extraction Kit (Axygen Biosciences, Union City, 129 CA, United States). To reduce PCR deviation, PCR reaction for one treatment was performed in triplicate.

#### MiSeq Processing and Data Analysis

The DNA samples were sequenced with an Illumina MiSeq 133 PE300 platform (Shanghai Majorbio Bio-pharm Technology Co., Ltd., China). To control sequencing quality, sequences with scores lower than 20 were discarded based on the QIIME quality control process (version 1.7.0). The operational taxonomic units (OTUs) at 97% similarity level were clustered using Usearch (vsesion 7.0^1^). The RDP Classifier (version 2.2^2^) in the Silva (Release128^3^) database was applied to perform sequence-level taxonomic assignment using confidence threshold of 70%. The non-metric multi-dimensional scaling (NMDS) was conducted using the Vegan software based on the unweighted uniFrac distance of genus.

#### Statistical Analysis

Fermentation parameters and microbial counts data were analyzed by two-way ANOVA for a 2 × 5 (treatment × storage periods) factorial arrangement of treatments by using the SPSS 19.0. Duncan’s multiple comparison was used for the means separation. Significant differences were declared when *P* < 0.05.

## Results

### Forage Characteristics

The characteristics of red clover forages upon ensiling are shown in Table 1. The DM, WSC, TN, FAA-N, and NPN of red clover were 21.0%, 109 g/kg DM, 29.3 g/kg DM, 65.2 g/kg TN, and 145 g/kg TN, respectively. The LAB, yeast and aerobic bacteria numbers were 6.21, 4.33, and 6.16 lg cfu/g FM, respectively. The FT event caused increases (*P* < 0.05) in FAA-N and NPN concentrations and TDD in the fresh material. The numbers of all enumerated microbes, particularly aerobic bacteria, increased (*P* < 0.05) in FT material compared with those in CK material.

**TABLE 1:** The characteristics of red clover forages upon ensiling.

**Items^1^**	**CK^2^**	**FT**	**SEM**	***P*-value**
DM (% FM)	21.0	20.7	0.22	0.536
WSC (g/kg DM)	109	97.1	6.62	0.884
TN (g/kg DM)	29.3	28.7	0.42	0.471
NPN (g/kg TN)	145	246	5.71	0.012
FAA-N (g/kg TN)	65.2	102	4.69	0.009
TTD (%)	14.4	70.0	3.47	0.002
LAB (lg cfu/g FM)	6.21	7.12	0.12	0.017
Yeast (lg cfu/g FM)	4.33	4.87	0.07	0.024
Aerobic bacteria (lg cfu/g FM)	6.16	7.68	0.26	0.002

*^1^FM, fresh matter; DM, dry matter; WSC, water-soluble carbohydrates; TN, total nitrogen; NPN, non-protein nitrogen; FAA-N, free amino acids N, TTD, tissue damage degree; LAB, lactic acid bacteria. ^2^CK, untreated control; FT, freeze-thawed red clover. SEM, standard error of means (*n* = 3, significant at *P* < 0.05).*

### Fermentation Parameters

As presented in Table 2, the pH, WSC concentrations, yeast and aerobic bacteria numbers declined, and fermentation products, such as lactic acid and acetic acid, and LAB counts increased with prolonged ensiling time. The propionic and butyric acids was below the detection limit. Despite higher (*P* < 0.05) LAB number was observed in FT material compared with CK material, the FT event seemed to result in a transitory lag in the fermentation process during the initial 1 day of ensiling, as indicated by higher (*P* < 0.05) pH and lower (*P* < 0.05) lactic acid concentrations in FT silage compared with those in CK silage at day 1. After 1 day of ensiling, more intense lactic fermentation was observed in FT silage, as indicated by lower (*P* < 0.05) pH and higher (*P* < 0.05) lactic acid concentration. At the end of ensiling, the lower (*P* < 0.05) pH and higher (*P* < 0.05) lactic acid concentrations indicated better fermentation quality of FT silage compared with CK silage.

**TABLE 2:** Fermentation parameters and microbial counts during ensiling.

**Treatment^1^**	**Day**	**pH**	**DM^2^(% FM)**	**WSC (g/kg DM)**	**NH_3_-N (g/kg TN)**	**Fermentation parameters (g/kg DM)**				**Microbial counts (lg cfu/g FM)**		
						**LA**	**AA**	**LA/AA**	**EOL**	**LAB**	**Yeast**	**Aerobic bacteria**
CK silage	1	4.74e	20.3bc	92.9c	39.4ab	17.9b	11.2a	1.57b	30.2b	6.21a	4.20cd	4.12d
	2	4.61d	18.8a	63.0b	54.4e	34.5c	12.4ab	2.55c	40.0d	8.12cd	4.10cd	4.02d
	4	4.46c	19.8abc	36.8ab	52.8cde	50.5d	16.1bcde	3.14d	38.0cd	7.95cd	3.71b	3.48b
	8	4.39c	19.2ab	18.2a	57.9ef	55.9e	17.9de	3.10d	37.2cd	8.30cd	3.54ab	3.14a
	30	4.39c	20.3bc	20.7a	64.8f	67.5f	23.2f	2.91d	35.0c	7.42b	3.44a	3.10a
FT silage	1	4.84f	20.8c	57.5b	36.9a	4.88a	13.9abc	0.35a	25.4a	7.22b	4.45e	3.98cd
	2	4.44c	19.4ab	58.8b	39.0ab	44.4d	15.2bcd	2.93d	27.9ab	8.81e	4.41de	3.87c
	4	4.29ab	19.0ab	53.1b	45.9bcd	60ef	17.1cde	3.51e	30.2b	8.04cd	3.92c	3.40b
	8	4.31b	19.5abc	38.3ab	45.5bc	67.9f	18.9e	3.59e	29.2ab	8.45d	3.41a	3.50bc
	30	4.22a	18.7a	21.6a	53.6de	78.2g	22.3f	3.53e	26.1ab	7.48b	3.54ab	3.67c
SEM		0.008	0.130	3.338	0.799	1.046	0.351	0.027	0.403	0. 210	0.173	0.073
*P*-value												
Treatment (T)		<0.001	0.450	0.595	<0.001	0.011	0.135	0.026	<0.001	<0.001	<0.001	<0.001
Day (D)		<0.001	0.019	<0.001	<0.001	<0.001	<0.001	<0.001	<0.001	<0.001	<0.001	<0.001
T × D		<0.001	0.073	0.454	0.136	0.005	0.583	<0.001	0.151	<0.001	<0.001	<0.001

*^1^CK, untreated control; FT, freeze-thawed red clover. ^2^FM, fresh matter; DM, dry matter; WSC, water-soluble carbohydrates; TN, total nitrogen; LA, lactic acid; AA, acetic acid; EOL, ethanol; LAB, lactic acid bacteria; cfu, coloning forming unit. SEM, standard error of means. Values in the same column with different letters differ significantly (*n* = 3, significant at *P* < 0.05).*

### Bacterial Diversity

**FIGURE 1:**
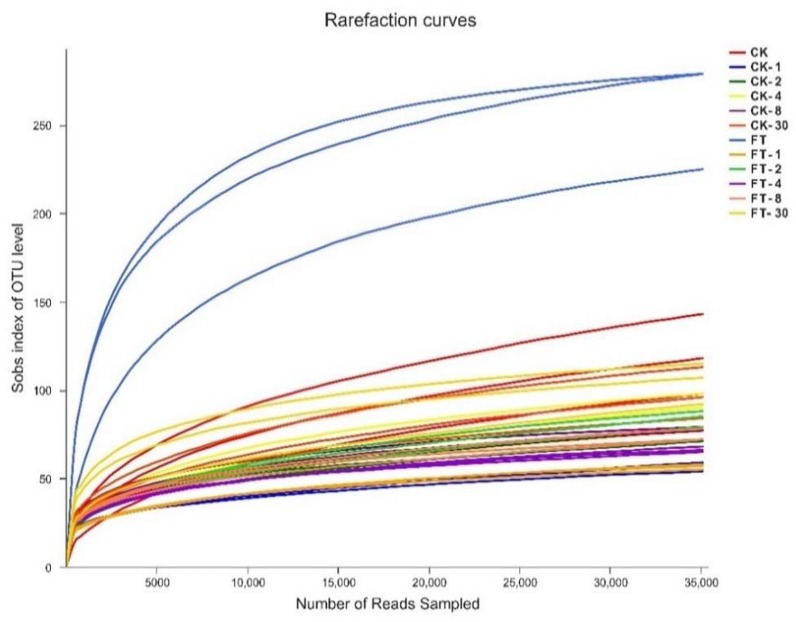
Rarefaction curves for all samples. CK, untreated control; FT, freeze-thawed samples. Arabic numbers following the treatment abbreviation indicate the ensiling day.

Approximately 1,837,106 valid sequences were obtained after removing unqualified sequences, with an average length of 449 bp per sequence for bacteria. These reads were clustered into 361 OTUs at 97% sequence identity. The rarefaction curve (Figure 1) and Good’s coverage (>99%, Table 3) indicated that sequencing depth had adequately captured most of the bacterial communities in all samples. Based on alpha diversity, the richness, and diversity of bacterial communities were evaluated in the two silages (Table 3). According to OTUs and Chao 1 index, the richness of the bacterial community dropped sharply after the onset of ensiling, especially in FT silage. Shannon index, a measure of the diversity based on the number and evenness of species, showed that among all material and silage samples the highest bacterial diversity was observed in FT material whereas lowest in CK material.

**TABLE 3:** Alpha diversity of bacterial diversity during ensiling.

**Treatment^1^**	**Day**	**Sequence number**	**OTUs^2^**	**Shannon**	**Chao 1**	**Coverage**
CK silage	0	56091	153	1.52	142	1.00
	1	48263	63.7	2.03	115	1.00
	2	58790	90.1	2.34	119	1.00
	4	56091	111	2.19	97.6	1.00
	8	43168	95.3	2.43	96.6	1.00
	30	41347	125	2.39	165	1.00
FT silage	0	58790	302	2.89	257	1.00
	1	51562	69.9	1.90	76.6	1.00
	2	44872	102	2.36	117	1.00
	4	58790	81.9	2.26	84.4	1.00
	8	41347	87.3	2.45	89.5	1.00
	30	48263	129	2.52	148	1.00

*^1^CK, untreated control; FT, freeze-thawed red clover. ^2^OTUs, number of operational taxonomic units; coverage, good’s coverage.*

**FIGURE 2:**
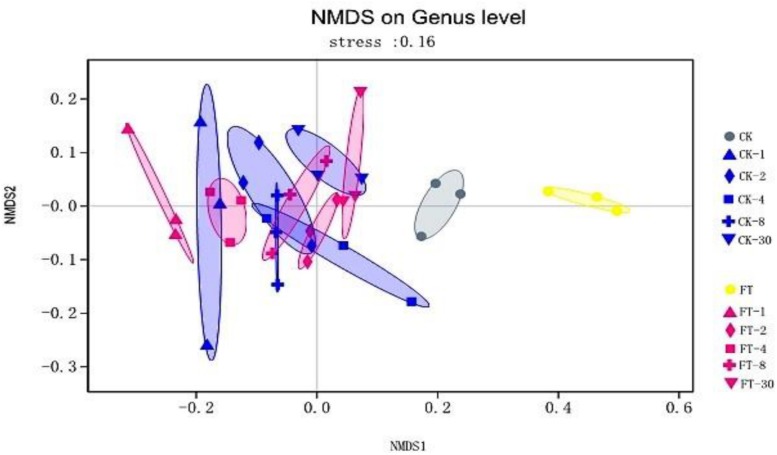
The dynamic variance of bacterial community at genus level shown by NMDS analysis. CK, untreated control; FT, freeze-thawed samples. Arabic numbers following the treatment abbreviation indicate the ensiling day.

The dynamic variance of bacterial community of the two silages during ensiling was observed by NMDS analysis. As shown in Figure 2, it can be seen that a clear separation and difference of bacterial communities in fresh materials from that in ensiled samples. Furthermore, the bacterial community of FT material was distinctive from that of CK material.

### Bacterial Community Dynamics

**FIGURE 3:**
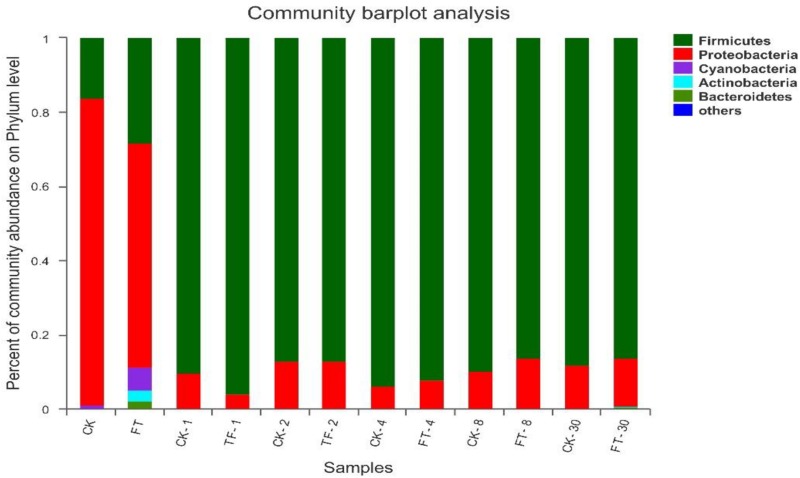
Dynamics of microbial community structure during ensiling at phylum level. CK, untreated control; FT, freeze-thawed samples. Arabic numbers following the treatment abbreviation indicate the ensiling day.

Three phyla (*Firmicutes*, *Proteobacteria*, *and Cyanobacteria*) were detected at high abundance in fresh red clover (Figure 3). Among them, *Proteobacteria* was the highest abundance phylum, accounting for 82.8% of the bacterial community. The FT event increased the relative abundances of *Firmicutes* from 16.2 to 28.2% and *Cyanobacteria* from 0.76 to 6.31%, and decreased the relative abundance of *Proteobacteria* to 60.5% in the ensilage material. In addition, two extra phyla (*Actinobacteria* and *Bacteroidetes*) were detected abundant in the FT material.

After the beginning of ensiling, *Firmicutes* quickly superseded *Proteobacteria in* CK silage to be the dominant phylum, and kept its dominant role (>87.2% of the bacterial communities) until the end of ensiling; the relative abundance of *Proteobacteria* phylum decreased sharply to <12.7%. Relative to CK silage, the FT event increased the relative abundance of *Firmicutes* and decreased the abundance of *Proteobacteria* at day 1 of ensiling, while the bacterial community structures of the two silages became similar after 2 days of ensiling. Phyla *Actinobacteria* and *Bacteroidetes* in the FT silage decreased to undetectable level after the onset of ensiling. However, their relative abundances became apparent again at the end of ensiling.

**FIGURE 4:**
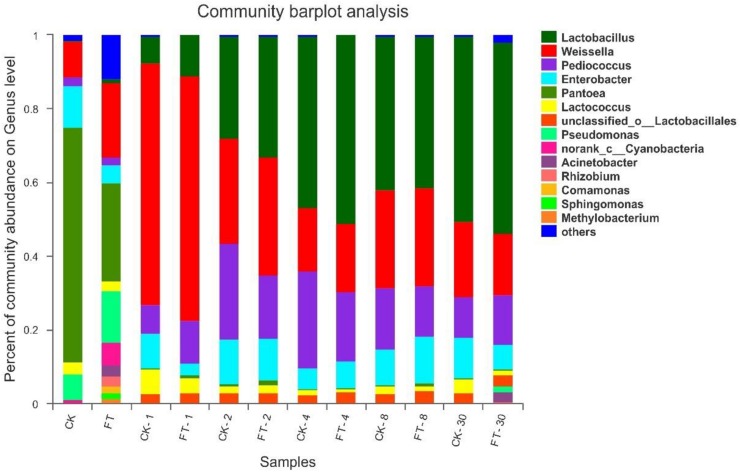
Dynamics of microbial community structure during ensiling at genus level. CK, untreated control; FT, freeze-thawed samples. Arabic numbers following the treatment abbreviation indicate the ensiling day.

To further understand the effects of FT event on microbial community dynamics during ensiling, bacterial community structures of the two silages were examined at genus level (Figure 4). The most prevalent genus in CK material was *Pantoea* (63.8%), followed by *Enterobacter* (11.3%), *Weissella* (9.72%) and *Pseudomonas* (7.04%), and *Lactococcus* (3.08%) and *Pediococcus* (2.25%). Compared with CK material, the abundant presence of various aerobic bacteria, such as *Pseudomonas* (14.1%), *Cyanobacteria* (6.40%), *Rhizobium* (2.86%), *Acinetobacter* (2.85%), *Comamonas* (1.93%), *Sphingomonas* (1.67%), *Methylobacterium* (1.24%) as well as some unclassified genera (11.9%), was found in FT material. Furthermore, it was observed that the relative proportions of genera *Pantoea* (26.6% vs. 63.8%) and *Enterobacter* (4.71% vs. 11.3%) deceased in the material microflora, whereas genera *Lactobacillus* (1.14% vs. undetectable level) and *Weissella* (20.3% vs. 9.72%) increased.

After the onset of ensiling, *Weissella* proliferated quickly, reaching to 65.7% of the bacterial community at day 1. However, its abundance decreased thereafter with prolonged ensiling time. The relative abundance of *Pediococcus* increased after the onset of ensiling and peaked (26.4%) at day 4 of ensiling. Along with the changes in *Weissella* and *Pediococcus* abundances, the relative abundance of *Lactobacillus* increased and became dominant after 4 days of ensiling. After the onset of ensiling, the all aerobic bacteria quickly decreased to undetectable levels. With respect to the *Pantoea*, they also dropped to marginal levels in the bacterial community. However, members of *Enterobacter* were still readily apparent throughout ensiling. Compared with CK silage, the FT event reduced *Enterobacter* (3.29% vs. 9.41%) at the day 1 of ensiling. In addition, it was observed that the relative proportions of *Lactobacillus* were always higher in FT silage compared with CK silage before 4 days of ensiling.

With prolonged ensiling time, microbiomes of the two silages were mostly constituted by LAB species and tended to be almost similar after 2 days of ensiling. However, genera *Acinetobacter* (2.74%) and *Pseudomonas* (1.65%) became apparent again in FT silage at the end of ensiling.

## Discussion

In practice, the FT event frequently occur before harvest or during silage making, leading to physical damages and vigorous growth of various bacteria in forage crops. It is known that microbial communities originally present on ensilage material play an important role in fermentation quality (Gharechahi et al., 2017). This means uncertainties of silage quality made from the damaged forages. The NGS technique has far superior resolution and is more adept at describing bacterial diversity than conventional methods (McAllister et al., 2018). Herein we compared the microbial community dynamics of silages made from FT-treated and untreated materials using NGS approach. As far as we know, this is the first report of the bacterial community in red clover silage.

### Changes in Forage Characteristics and Fermentation Parameters After FT Event

As indicators of protein hydrolysis (Li et al., 2018), the increased FAA-N and NPN concentrations suggested larger extent of protein hydrolysis in the FT material upon ensiling. In living plant, extensive degradation of plant protein is prevented by cellular compartmentation (Carrión et al., 2014). The FT event disrupted the cellular compartmentation, thereby increasing the exposure of forage proteins to plant proteases (Grabber and Coblentz, 2009). The greater proteolysis extent confirmed the significant physical changes in plant cell after the FT event, consistent with the observation of increased TDD of the FT material.

Despite the FT procedures were conducted under conditions where as possible as to avoid soil and water contamination, the numbers of all enumerated microbes, particularly aerobic bacteria, and increased significantly in the FT material compared with the CK material upon ensiling. The increased microbial counts were assumed to link the increased nutrients release that favored proliferation of indigenous microbes and likely some airborne bacteria (Leifert et al., 1994).

Legume forages are generally regarded as difficult to ensile because of high buffering capacity and low sugar content (McDonald et al., 1991). Clostridial fermentation is easy to occur particularly when legume forages are ensiled at high moisture (Dong et al., 2017). In this study, butyric acid, the product of clostridial fermentation, were absent in all silages. These observations, coupled with the low terminal pH and dominance of lactic acid, indicated satisfactory fermentation quality of the red clover silages. The quality fermentation might be attributed to the high WSC content (109 g/kg DM) that encouraged lactic fermentation. This was in accordance with numerous studies where fermentable sugar contents were observed to be low in other legume forages, such as alfalfa, whereas usually high in red clover (Raguse and Smith, 1966; Owens et al., 1999).

Under anaerobic conditions, LAB ferment soluble carbohydrates to organic acids, mainly lactic acid, results in decline in silage pH (Pahlow et al., 2003). The fast dominance of LAB in silage microflora is crucial for the resultant fermentation. Yang et al. (2019) previously showed that inoculation of LAB to sterilized material than to non-sterilized material resulted in a faster predominance of LAB. This leads to the deduction that complex microbial composition of FT material may increase the difficulty of LAB to outgrow in the microflora at the onset of ensiling and explained the initial short lag in lactic fermentation of FT silage compared with CK silage. At the end of ensiling, better fermentation quality was observed in FT silage. The plausible explanation could be that the FT event increased the leakage of plant cellular contents, making plant nutrients more readily available for LAB growth (Charmley et al., 1997). However, information obtained by these conventional fermentation parameters was very limited, increasing the need for deeper insights into microbial community dynamics during the ensiling.

### Changes in Bacterial Diversity and Composition After FT Event

Bacteria present in fresh forage crop and silage can be simply classified into three groups based on their contributions to silage quality and abilities to thrive in anerobic environment: LAB, which are desirable bacteria contributing to the production of lactic acid and pH decline; undesirable facultative aerobes, which compete with LAB for nutrients during ensiling and contribute little to the pH decline; strict aerobes, which are also undesirable bacteria whereas remain active only before oxygen is depleted in silo (McDonald et al., 1991).

Our results showed that *Proteobacteria* and *Firmicute* were two most abundant phyla in fresh red clover, comprising 99.2% of the microflora. After the FT event, the relative abundance of *Firmicute* increased from 16.2 to 28.2%. The increased *Firmicute* was attributed to increased abundance of *Weissella*, which belong to *Firmicute*. *Weissella*, an obligatory heterofermentative LAB species, are major components of the microflora in various types of forage crops (Cai et al., 1998; Yang et al., 2019). They are initial LAB population that play a key role in the fast acidification at the onset of ensiling. The enrichment of *Weissella* in FT material indicated the beneficial effects of FT event on the silage fermentation and was consistent with higher numbers of LAB in FT material. The accurate reason for the increased *Weissella* abundance is unclear, probably associated with the liberation of chemical signals from the damaged plant, such as amino acids, that play as a specific stimulus to growth of LAB (Barnett, 1952). In addition, it could be expected that the release of plant juice from the damaged plant may also help LAB spread during handling (Stirling and Whittenbury, 1962).

*Lactobacillus*, *Pedicoccus*, and *Weissella* are considered as the 3 most predominant LAB genera responsible for driving lactic fermentation during ensiling (Pang et al., 2011; Liu et al., 2019). After the onset of ensiling, significant shift in bacterial community from *Proteobacteria* to *Firmicute* could be attributed to the increased abundances of genera *Lactobacillus*, *Weissella* and *Pediococcus*, which flourished in the environmental conditions developed during ensiling (Keshri et al., 2018). Furthermore, in this study the intensive sampling points revealed a dominance succession from *Weissella* and *Pediococcus* to *Lactobacillus* in lactic fermentation. It is known that *Weissella* is an early colonizer (Graf et al., 2016), and *Pediococcus* contributes to initial decline in silage pH, creating an environment suitable for the development of *Lactobacillus* (Yang et al., 2019). These two LAB genera are not as tolerant as *Lactobacillus* to acidifying environment and thus active only during the early stages of ensiling. The follow-up lactic acid production mainly depends on *Lactobacillus*, which become active and grow vigorously as pH decreases (Cai et al., 1998). Compared with CK silage, it was observed that the relative proportions of *Lactobacillus* were always higher in FT silage before 4 days of ensiling. This probably explained the more intense lactic fermentation. The higher proportions of *Lactobacillus* were assumed to be related with the enrichment of *Weissella* in the FT material that created an initial acid environment favorable for the following *Lactobacillus* development (Cai et al., 1998).

This study showed that *Pantoea* and *Enterobacter* were two major facultative aerobe genera present in fresh red clover microflora. *Pantoea* and *Enterobacter* have been reported to be present in various forages, such as alfalfa (Ogunade et al., 2018) and soybean (Ni et al., 2017b). Their populations are affected by environmental factors, such as climate, geographical location, and type of fertilizer used (Yang et al., 2019). The FT event did not favor their growth; in contrast, it deceased their relative proportions in the fresh material. The deceased *Pantoea* and *Enterobacter* might have two reasons. First, increased *Weissella* abundance exhibited an inhibitory effect on other bacteria by producing bacteriocin (Papagianni and Papamichael, 2011); Second, vigorous multiplication of other bacteria, such as aerobic bacteria, reduced the relative proportions of *Pantoea* and *Enterobacter* in the microbial community.

After the beginning of ensiling, drops in *Pantoea* abundances reflected their high sensitivity to pH decline (Ogunade et al., 2018). However, the members of *Enterobacter* were apparent throughout the ensiling, likely because of the presence of some acid-tolerant *Enterobacter* species (McGarvey et al., 2013). Compared with CK silage, the FT silage exhibited reduced relative abundance of *Enterobacter* at day 1 of ensiling, probably owing to its relative lower abundance in FT material. From this aspect, the facultative aerobes might not be the threat for the silage quality made from the FT-damaged forages. However, facultative aerobes are abundantly present in various environmental sources, such as soil and animal manure (Ogunade et al., 2018). Under field conditions, the altered physical features, induced by the FT event, may increase the risk of the damaged forages being contaminated with the facultative aerobes from the environment, which also highlights the importance of hygienic quality during harvesting and processing.

In the current experiment, aerobic bacteria flourished after the FT event, contributing to the high bacterial richness, and diversity in the FT material. These aerobic bacteria were supposed to compete with the LAB for nutrients during the initial aerobic phase of ensiling (Dong et al., 2017), explaining the initial short lag in lactic fermentation of the FT silage. As expected, due to the achievement of acidic and anaerobic environment in silage, the aerobic bacteria all fell to undetectable levels after the onset of ensiling. The decreases in abundance of aerobic bacteria could be responsible for the drops in bacterial richness and diversity in FT silage after the onset of ensiling. It is worth noting that genera *Acinetobacter* (2.74%) and *Pseudomonas* (1.65%) became apparent again in FT silage at the end of ensiling. *Acinetobacter* and *Pseudomona* are aerobic, non-fermenting bacteria, which can be found in different environments (Palleroni, 2010; Kampfer and Glaeser, 2012). The roles of these bacteria in silage have not been extensively studied. Although they are supposedly absent in silage (Li and Nishino, 2011), some species are able to survive in anaerobic environment in the presence of acetate as a substrate (Fuhs and Chen, 1975). When silages are exposed to air, these survived aerobic bacteria may proliferate firstly if silage pH is not low enough to suppress them, leading to aerobic deterioration of the silage (Liu et al., 2013). Liu et al. (2019) previously found that *Acinetobacter* was the dominated spoilage organisms in aerobically deteriorating barley silage. The survival of these bacteria also increases the evidence that the microbes responsible for aerobic deterioration after silo opening are indigenous to the silage rather than aerial-borne invaders.

## Conclusion

*Weissella* played an important role in the initiation of lactic fermentation in red clover silage. The FT event promoted the development of *Lactobacillus* during ensiling, and reduced the relative abundances of *Pantoea* and *Enterobacter* at the onset of ensiling, indicating the beneficial effects on fermentation quality. However, due to vigorous growth of aerobic bacteria in FT material, *Acinetobacter*, and *Pseudomonas* became apparent again in FT silage at the end of ensiling. This reveals that the control of aerobe revitalization could be a challenge for the silage quality made from the freeze-damaged forages. In fact, knowledges obtained in this study might be applicable not only to those circumstances where forages are subjected to freezing damages and also to circumstances where forages are imposed by mechanical damages (for e.g., rolling, crushing, and maceration).

## Author Contributions

TS designed the experiments. JL, LC, SW, and ZD conducted the experiments. ZD analyzed the data and wrote the manuscript.

## Conflict of Interest Statement

The authors declare that the research was conducted in the absence of any commercial or financial relationships that could be construed as a potential conflict of interest.

## Footnotes

1 http://drive5.com/uparse/

2 http://sourceforge.net/projects/rdp-classifier/

3 https://www.arb-silva.de/
